# Proactive control of attention in math-anxious individuals

**DOI:** 10.1007/s00426-022-01750-3

**Published:** 2022-10-21

**Authors:** Àngels Colomé, María Isabel Núñez-Peña, Belén González-Gómez

**Affiliations:** 1 Section of Cognitive Processes, Department of Cognition, Development and Educational Psychology, Faculty of Psychology, University of Barcelona, Passeig Vall d’Hebron, 171, 08035 Barcelona, Spain; 2 Institute of Neurosciences, University of Barcelona, Barcelona, Spain; 3 Section of Quantitative Psychology, Department of Social Psychology and Quantitative Psychology, Faculty of Psychology, University of Barcelona, Barcelona, Spain; 4 Institut de Recerca Sant Joan de Déu, Esplugues de Llobregat, Spain

## Abstract

Attentional control deficit has been proposed as one of the reasons for lower arithmetical performance in people with high math anxiety (HMA). Previous research trying to discern whether this deficit concerned proactive or reactive use of attentional control has been criticised because the methodologies used were mostly suited to investigating reactive control only. The aim of this study was to investigate proactive control in HMA individuals in a classical Stroop task. Twenty HMA and 20 low math-anxious individuals (LMA) named the ink colour in which congruent and incongruent colour words as well as X strings (neutral condition) were presented. The HMA group was slower than their LMA peers in the congruent and incongruent conditions only. Furthermore, HMA individuals showed a higher interference effect. Last, only LMA participants showed a facilitatory effect of the congruent condition. These results are interpreted as indicating the presence in the HMA individuals of a task conflict between the task to perform (ink naming) and an irrelevant task triggered by the stimuli (word reading). Task conflict is evident only when proactive control, responsible for maintaining the current goals, is too weak to solve the competition between tasks. Therefore, this study confirms that HMA individuals find it difficult to implement attention proactively.

## Introduction

Math anxiety is frequently described, using the classical definition given by Richardson and Suinn (1972), as a feeling of tension and apprehension that some people experience when they have to manipulate numbers or solve mathematical problems, in academic or daily situations. Many studies have been devoted to investigating this phenomenon, mainly because math anxiety has a higher prevalence than initially suspected (Organization for Economic Co-operation and Development [OECD], 2013) and is often related to worse mathematical performance (e.g., Ashcraft & Kirk, 2001). An approach to explain the effects of math anxiety on performance that is receiving increasing interest involves the use of Attentional Control Theory (ACT hereafter, Eysenck et al., 2007).

ACT claims that anxiety impairs the efficiency of central executive functions related to attentional control: inhibition, understood as the capacity to prevent irrelevant stimuli or responses from harming performance, and shifting, or the capacity to flexibly change the allocation of attention to the current relevant task or stimuli (Eysenck & Derakshan, 2011). Our aim in this study was to investigate the inhibition capacities of highly math-anxious individuals.

In the case of individuals with high math anxiety (HMA), this deficit in attention control might lead them to pay excessive attention to their ruminations on their poor performance or abilities, or to bias attention towards external mathematically related stimuli, which are perceived as threatening (Lyons & Beilock, 2012). This bias would leave fewer attentional resources available for the relevant task and be detrimental to their performance. Attentional bias towards mathematically related stimuli was shown, for instance, by Suárez-Pellicioni et al. (2015) in an emotional Stroop task (see also Georges et al., 2016; Hopko et al., 1998; Núñez-Peña et al., 2021 and Passolunghi et al., 2016, for evidences of HMA’s attentional deficit in inhibition).

Recently, there has been an attempt to further specify the inhibitory control component impaired in HMA using the distinction of two modes of cognitive control proposed by the Dual Mechanism of Control Theory (DMC, Braver, 2012). Proactive control is a goal-driven mechanism that consists of applying attention in a sustained way to ensure that our behaviour adjusts to our goals. It helps prevent interference before it appears. *Reactive control* is stimulus-driven, occurring only when an interference is detected, and acts as a late correction mechanism by reactivating the task-goals. According to Braver (2012), proactive and reactive control mechanisms might be semi-independent and could be engaged simultaneously, but it is more likely that one of them is favoured over the other depending on the individual and task characteristics. Proactive control is preferred when interferences are frequent and predictable, but it needs goal-directed information to be kept in the working memory, which is an effort that individuals might want to assume only if its consequences are valuable enough. Furthermore, when working memory resources are limited, reactive control might have to be used instead. Braver (2012) claimed that this might be the case in trait (or state) anxiety, because some of the working memory resources are focused on coping with the internal ruminations or threatening external stimuli (e.g. Fales et al., 2008; Yang et al., 2018).

Regarding math anxiety, in Suárez-Pellicioni et al. (2014) we conducted an event-related brain potential (ERP) study in which HMA and low math anxious (LMA) participants performed a numerical Stroop task, where they had to choose the largest numerical magnitude while ignoring physical size. We analysed two effects: congruity effect in the current trial and conflict adaptation, i.e., the interaction between the congruency of the previous trial and the current one. HMA participants showed more interference between physical and numerical magnitudes than LMA individuals, suggesting that HMAs are more susceptible to distraction because they apply attention reactively. As for ERPs, only HMA participants showed a reduction in Conflict Sustained Potential (Conflict S-P) after incongruent compared with congruent trials. Since the amplitude of Conflict S-P has been positively related to the magnitude of the conflict, we concluded that HMA participants had only applied reactive attention after an incongruent trial. In contrast, LMA participants showed no differences in their Conflict S-P after congruent and incongruent trials, as would be expected if they had engaged their attention proactively. Altogether, our results confirmed the prediction of the DMC theory (Braver, 2012) that math anxiety prevents the HMA participants from using proactive control and forces them to rely on reactive mechanisms.

This conclusion has recently been discussed by Van den Bussche et al. (2020), who considered that the paradigm used by Suárez-Pellicioni et al. (2014) might be ill-suited for measuring proactive attention. Given that there was an equivalent proportion of congruent and incongruent trials, interference was unpredictable; according to Van den Bussche et al. (2020), conflict anticipation in this case would not be optimal and reactive control would be preferred. Furthermore, they claimed that since conflict adaptation is an index of reactive recruitment of attention, making conclusions about proactive attention might be too much of a leap.

Van den Bussche et al. (2020) used an arrow flanker task with the aim of assessing both reactive and proactive control. Their participants had to decide the direction of a central arrow and ignore the direction of distractor arrows at its side that went in the same (congruent) or opposite (incongruent) direction. In the mostly congruent (MC) block, only 20% of the trials were incongruent and using proactive attention would have made no sense, because the interferences to which the subjects had to react were scarce and proactive mechanisms consume considerable resources. Therefore, slower latencies were expected in the unanticipated incongruent trials and a larger congruency effect was predicted. In the mostly incongruent (80% trials) block (MI), use of proactive control and a smaller congruency effect were expected.

The results showed that congruency effect increased with math anxiety, but only in the MC block. Furthermore, the largest effects of math anxiety were found in the incongruent trials of the MC block. Van den Bussche et al. (2020) concluded that math anxiety impairs reactive control because in the intervals between interference, anxious people tend to distribute their attention widely; when attention has to be quickly relocated, they would be more vulnerable to distraction by non-relevant stimuli. As for the lack of anxiety effects in blocks assumed to require proactive control, this contradicts DMC predictions (Braver, 2012) and contrasts with previous findings on other types of anxiety (e.g. Fales et al., 2008; Yang et al., 2018): Van den Bussche et al. (2020) suggested that their task might have been too easy and required few attentional resources, such that the reduced memory capacity assumed in math-anxious individuals would have been enough.

In summary, Braver’s DMC Theory (2012) predicts that anxiety might impair proactive control mechanisms. Previous studies on math anxiety (Suárez-Pellicioni et al., 2014; Van den Bussche et al., 2020) have found contradictory results and their conclusions regarding proactive control have been questioned based on methodological issues. Given these antecedents and the fact that Braver (2012) raised the possibility that proactive and reactive mechanisms were semi-independent, it seemed appropriate to investigate proactive control per se instead of making conclusions about it based on results obtained in tasks more suited to investigating reactive mechanisms. Hence, our aim was to investigate proactive control in people with HMA.

To do so, we made use of a classical Stroop task in which we measured Stroop facilitation. In the Stroop task, participants are presented with colour words (and sometimes control nonwords) displayed in different ink colours and have to name the colour in which words have been presented while ignoring the meaning of the printed word, even though it is automatically processed. Most studies focus on the interference effect, that is the longer reaction times (RTs) for incongruent trials (e.g. saying “blue” when faced with the word “green” printed in blue) compared to neutral ones (XXXX string printed in blue). Interference is thought to be caused by an information conflict: although we pay attention to the colour, the meaning of the word is also processed and competes for selection. A facilitation effect is also sometimes found: participants are faster when saying “blue” faced with the word “blue” printed in this colour than when faced with a blue string of Xs. However, this effect is usually less robust and sometimes even disappears or transforms in reverse facilitation (RF), with neutral stimuli leading to faster responses than congruent ones. This has led some authors (Godfarb & Henik, 2007; see also Kalanthroff et al., 2018) to propose that the facilitation effect might be hiding a task conflict: under normal circumstances task conflict would not be behaviourally evident, but it might become clear in some situations or populations with difficulties regarding attentional control. Indeed, neuroimaging studies (e.g. Bench et al., 1993; Carter et al., 1995) showed that the anterior cingulate cortex, a brain area related to conflict monitoring, is more active in congruent than in neutral stimuli. This conflict has been interpreted as being caused by stimuli activating two competing tasks, because reverse facilitation is observed when nonwords are used as neutral stimuli but not when a noncolour word is used instead (e.g. Goldfarb & Henik, 2007). Kalanthroff and colleagues (see 2018, for a review) suggested that the conflict between reading the word and naming the colour that would slow RTs in congruent trials would not exist in the nonword neutral ones. They proposed the PC-TC (proactive control, task-conflict) model, related to Braver’s DMC model.^1^ According to them, a stimulus can trigger a response that is strongly associated with it (for instance, reading a word) but is not relevant at that moment, thus creating competition between two task sets. Nevertheless, task sets can also be activated proactively, to reach the current goals. If proactive control functions properly, task conflict will not be noticeable and facilitation will be observed, because of the coincidence of the information activated by the two tasks. However, when proactive control is not working efficiently (e.g. when working memory resources are occupied in a concurrent task, Kalanthroff et al., 2015), bottom-up activation might be behaviourally evident and reverse facilitation observed. According to Kalanthroff et al. (2018), people with anxiety disorders might also show reduced proactive control, because some of their working memory resources are devoted to coping with task-irrelevant emotions or ruminations (see Kalanthroff et al., 2016, for a study on trait anxiety).

In our case, we used a colour Stroop task and presented our HMA and LMA participants with congruent, incongruent and neutral nonword conditions. Our prediction was that if the proactive control capacity of HMA individuals is disrupted, they would show more task conflict than their LMA peers, which would translate into a slowdown in both the congruent and incongruent trials, but not in the neutral ones. Consequently, we expected HMA participants to show a larger interference (incongruent > neutral) effect and a smaller or even reverse facilitation (congruent < neutral) effect.

## Methods

### Participants

Forty students from the University of Barcelona took part in this experiment. They were selected on the basis of their extreme scores in the Shortened Mathematics Anxiety Rating Scale (sMARS) (Alexander & Martray, 1989) and were part of a larger sample assessed for a longer project during an introductory psychology course. Twenty participants were included in the low math anxiety group (LMA) because they scored below the first quartile in the sMARS scores of the larger sample (mean = 43.5, SD = 6.4, range = 25–53). The high math anxiety group (HMA) was formed by 20 participants scoring above the third quartile (mean = 86.6, SD = 10.8, range = 77–122). As expected, the math anxiety scores of the two groups differed significantly, *t* (38) = 15.40, *p* < 0.001.

In contrast, both groups were comparable in terms of age, *t*(38) = 1.49, *p* = 0.14, and gender distribution (11 females in each group). Lastly, given that trait anxiety has been shown to affect cognitive control (e.g., Kalanthroff et al., 2016), we ensured that the two groups did not differ in their scores on the Trait Anxiety Scale (Spielberger et al., 1983), *t*(38) < 1. All students signed an informed consent and received monetary compensation. This experiment is part of a research project that has been approved by the Ethics Committee of the University of Barcelona. See Table 1 for more information on participants.

**Table 1:** Means and SD (in brackets) for math anxiety, trait anxiety, and age as well as gender distribution (number of men) for the high (HMA) and low (LMA) math anxiety groups

	Math anxiety	Trait anxiety	Age	Gender
LMA	43.50 (6.38)	21.05 (6.23)	22.40 (2.44)	9
HMA	86.60 (10.77)	21.75 (7.31)	23.65 (2.85)	9

### Materials

#### Screening

##### Shortened Mathematics Anxiety Rating Scale (Alexander & Martray, 1989)

The Spanish version of this test was used (Núñez-Peña et al., 2013). It consists of 25 situations that can cause math anxiety and requires participants to rate on a five-point Likert scale, 1 (no anxiety)—5 (high anxiety), the level of anxiety caused by each item. The Spanish version has strong internal consistency (Cronbach’s alpha = 0.94) and high 7 week test–retest reliability (intra-class correlation coefficient = 0.72).

##### State–Trait Anxiety Inventory (STAI) (Spielberger et al., 1983)

Only the trait anxiety scale (STAI-T) was used. Participants must answer how they feel in “general” using a four-point Likert scale ranging from 0 (almost never) to 3 (practically always), on 20 items describing different emotions. The Spanish version of the scale (Spielberger et al., 2008) was used: it has excellent internal consistency (Cronbach’s alpha = 0.95), and adequate 20-day test–retest reliability with college students (*r* = 0.86).

#### Experimental session

Participants were asked to name the colour in which a stimulus was displayed on the computer screen while ignoring its meaning. Responses could be the Spanish words for blue (*azul*), red (*rojo*), green (*verde*) and yellow (*amarillo*). Each ink colour was presented under three different conditions: in the *congruent* one, the colour was used to display its own colour name. In the *incongruent* one, any of the other three colour names was presented. In the *neutral* condition, a string of four Xs was displayed. There were 240 trials in which each ink colour was paired in the same number of trials with each colour word and with the neutral sequence, leading to 20% congruent, 20% neutral, and 60% incongruent trials. The fact that the majority of trials were incongruent is considered to favour the use of proactive control (e.g. De Pisapia & Braver, 2006). Three blocks were created with the three conditions equally distributed in each of them. Trials within each block were randomised. A training block of nine trials preceded the experiment.

Each trial had the following structure. A white asterisk appeared on a black screen for 250 ms and was replaced by a blank of the same duration. Immediately after, a colour word or a string of Xs (Arial, 40) were displayed until the participant named their colour or for a maximum of 2000 ms. A blank screen lasting 500 ms was displayed between trials. Vocal responses were recorded from the onset of the stimuli. E-prime 2 (Psychology Software Tools Inc., Sharpsburg, PA, USA) was used to present the stimuli and record the answers’ latencies.

## Results

**Fig. 1:**
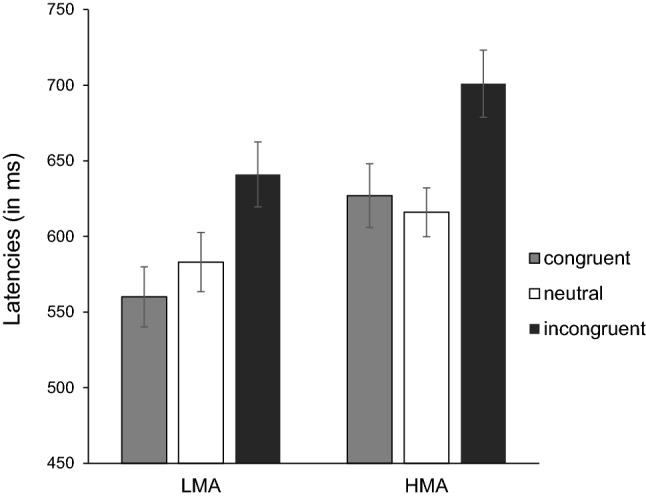
Mean latencies for each experimental condition for the low math-anxious (LMA) and high math-anxious (HMA) groups. Bars denote standard errors

2.65% of the responses were removed before the analyses because they contained verbal disfluencies or nonverbal sounds that triggered the voice key. An ANOVA was conducted on the median latencies of correct trials, with condition (congruent, incongruent, and neutral) as the within-subject variable and group (HMA and LMA) as the between-subject factor. See Fig. 1 for the means and standard errors of latencies for the two groups in each experimental condition.

There was a main effect of condition, *F*(2,76) = 92.96, *p* < 0.001, *η*_*p*_^*2*^ = 0.71, and a significant condition x group interaction, *F*(2,76) = 4.11, *p* = 0.02, *η*_*p*_^*2*^ = 0.10, while the difference between groups was only marginally significant, *F*(1,38) = 3.76, *p* = 0.06, *η*_*p*_^*2*^ = 0.09. Low math-anxious participants were significantly faster than their highly math-anxious peers in the congruent trials, *t*(38) = 2.33, *p* = 0.02, and answered marginally faster in the incongruent condition, *t*(38) = 1.93, *p* = 0.06, but the two groups did not differ in the neutral condition, *t*(38) = 1.29, *p* = 0.20. Given that we had specific predictions for group differences in the facilitatory and interference effects, we ran new ANOVAs in which we analysed them separately. Regarding the facilitation effect, congruent and neutral stimuli did not differ, *F* < 1. However, there was a marginal effect of math anxiety, *F*(1,38) = 3.57, *p* = 0.06, *η*_*p*_^*2*^ = 0.08, as well as a significant condition x group interaction, *F*(1,38) = 8.82, *p* = 0.005, *η*_*p*_^*2*^ = 0.18. When looking at each group separately, standard facilitation for congruent trials was found in the low math-anxiety group, *t*(19) = 3.11, *p* = 0.006. There was a trend towards reverse facilitation for HMA participants (627 vs. 616 ms in the congruent and neutral trials, respectively), although it did not reach significance, *t*(19) = 1.28, *p* = 0.21.

Regarding interference, a main effect of condition was found, *F*(1,38) = 117.14, *p* < 0.001, *η*_*p*_^*2*^ = 0.75, as well as a significant condition x group interaction, *F*(1,38) = 4.07, *p* = 0.05, *η*_*p*_^*2*^ = 0.10. Although both groups showed an interference effect (*t*(19) = 9.04, *p* < 0.001 and *t*(19) = 7.35, *p* < 0.001, for LMA and HMA groups, respectively), the size of this effect was larger for the HMA group (*t*(38) = 2.01, *p* = 0.05, 58 vs. 85 ms). Latencies of the two groups did not differ, *F*(1,38) = 2.84, *p* = 0.10, *η*_*p*_^*2*^ = 0.07.

Accuracy was in general very high (99% for the congruent and neutral conditions), although it decreased slightly in the incongruent trials (96%). The same ANOVA as used in the latencies analysis was performed and it confirmed a significant effect of condition, *F*(2,76) = 21.75, *p* < 0.001, *η*_*p*_^*2*^ = 0.36. Accuracy was lower for incongruent than for congruent, *t*(39) = 5.18, *p* < 0.001, or neutral trials, *t*(39) = 4.56, *p* < 0.001, and the latter two conditions did not differ, *t*(39) = 1.60, *p* = 0.11. Neither the group effect nor the interaction were significant (all *ps* > 0.20), and accuracy was not further analysed.

## Discussion

It has been suggested that individuals suffering from math anxiety might have a deficit in attentional control that would make them more vulnerable to distractions. Recent studies on attention have made a further distinction between proactive and reactive attention. Anxiety has been named as one of the factors that might weaken proactive control but previous attempts at studying it in math anxious populations have been criticised for not using the most suitable methods. On this occasion, we used a classical Stroop task and measured task conflict, as it is a widely accepted method for investigating proactive control. We found that our two anxiety groups behaved similarly in the neutral condition. In contrast, HMA individuals were slower than their LMA peers in the congruent and (marginally) in the incongruent trials, that is, the conditions in which a conflict arises between the task to perform (naming the colour) and the reading task strongly associated with the colour words. Interference was found in both groups, but HMA participants suffered it to a greater degree. Last, facilitation was found in the LMA group only. Altogether, our data points toward the existence of a task conflict in math anxious individuals, and therefore, an attenuation in their proactive implementation of attentional control.

Our results fit nicely with the predictions of the ACT (Eysenck et al., 2007) and PC-TC model (Kalanthroff et al., 2015), this one based on the Dual Mechanism of Control model (Braver, 2012). The ACT predicts that anxiety will impair attentional control by biasing the attentional system towards bottom-up, stimulus-driven processing. In our case, the activation of the reading task by the presentation of a word would have entered into competition with the required colour-naming task.

DMC (Braver, 2012) specifies that impairment will mainly affect the use of proactive attention, that is, the anticipatory maintenance of relevant goals to ensure that the pertinent processing takes place. Although Braver and collaborators used the Stroop paradigm to test their hypotheses (e.g. Bugg et al., 2011), they never measured task conflict. Hence, we based our predictions on the PC-TC model, which extends DMC to explain task conflict due to control variability. The PC-TC model implements proactive control as a form of top-down activation that ensures that the proper task is selected. In the case of anxious individuals, this activation would not be enough to solve the competition between the two tasks (reading/naming the colour) activated by the stimuli, leading to a slowdown in the responses. Conflict would be solved by applying attention reactively. DMC and PC-TC models differ in the conditions under which reactive attention comes into play. In the case of DMC, reactive attention is triggered when an informational conflict (i.e. a conflict between two activated responses) is detected and hence this model cannot explain why HMA individuals also show longer latencies in congruent trials, where there is no informational conflict. In contrast, according to the PC-TC model, reactive attention also comes into play when proactive attention is too weak to solve the competition caused by the bottom-up activation of tasks by the stimuli, and helps bias competition towards the relevant task.

We cannot conclude without mentioning a puzzling finding that is repeatedly reported in the literature and was replicated here. It is usually considered that anxious individuals behave differently from their peers because feelings and ruminations related to their anxiety deplete some of the central executive resources and prevent them from applying attention proactively. Yet, HMA individuals have shown a deficit in attentional control in many tasks that did not contain mathematical or numerical stimuli, and in principle, should not have caused anxiety: reading non-mathematical words (Hopko et al., 1998), deciding the colour of an arrow (Georges et al., 2016), an arrow flanker task (Van den Bussche et al., 2020) or a colour Stroop test as in the current study. Importantly, we can rule out the possibility that group effects were caused by general (trait) anxiety because we ensured that it did not differ between them. One possible interpretation, already suggested by Hopko et al. (1998), would be that math anxious individuals suffer from a domain-general deficit in attentional control. Another alternative, proposed by Van den Bussche et al. (2020), is that despite not having any math-related content, the tasks used in these studies would require processing that is typical of mathematical tasks. Since HMA individuals might have felt tense when performing such mathematical tasks in the past, they would extend these negative feelings to other non-mathematical occasions for which the same processes were necessary. A final possibility that cannot be completely ruled out is that poor attentional control precedes math anxiety and helps increase it by worsening math performance. De Agostini (2020), for instance, found that inefficient inhibition was a significant longitudinal predictor of math anxiety. Although not within the scope of this paper, discerning between these possibilities in the future will be crucial to devise proper ways to improve attentional control in people with math anxiety.

In summary, HMA individuals showed the presence of task conflict in conditions in which distractor words were presented. Given that task conflict is considered as an indicator of a deficit in proactive control, our data confirm that HMA individuals have difficulty applying attention to achieve their goals in an anticipatory and sustained way.

## Data Availability

The dataset generated and analysed during the current study is available in the OSF: https://osf.io/nwphf/?view_only=13bc37be3309432da76e03e19d7bafb7.

## Footnotes

1 Braver’s (De Pisapia & Braver, 2006) simulation of attentional control in the Stroop task used noncolour words (e.g. DOG) as the neutral condition, and therefore could not investigate task conflict.
